# Length of stay, costs, and complications in lumbar disc herniation surgery by standard PLIF versus a new dynamic interspinous stabilization technique

**DOI:** 10.1186/s13037-017-0141-1

**Published:** 2017-11-23

**Authors:** Manuel Segura-Trepichio, David Candela-Zaplana, José Manuel Montoza-Nuñez, Antonio Martin-Benlloch, Andreu Nolasco

**Affiliations:** 1 Departamento de Cirugía Ortopédica y Traumatología, Hospital Universitario del Vinalopó, Alicante, Spain; 2 Departamento de Cirugía Ortopédica y Traumatología, Hospital Universitario Dr Pesset, Valencia, Spain; 3 Unidad de investigación para el análisis de las desigualdades en salud y la mortalidad FISABIO-UA, Universidad de Alicante, Alicante, Spain; 4 Department of Orthopedic Surgery, Vinalopó University Hospital, Elx/ Elche, 03203 Alicante, Spain

**Keywords:** Lumbar disc herniation, Discectomy, Lumbar fusion, Length of stay, In-hospital costs, Surgical safety, Readmission, Reintervention, Complications, Prospective cohort study

## Abstract

**Background:**

The number of lumbar spine surgeries has been increasing during the last 20 years, which also leads to an increase in hospital costs and complications related to surgery. Therefore, there is a greater concern about the costs and safety of the techniques and implants used.

**Methods:**

Patients (aged from 18 to 50 years) presenting with lumbago /sciatica (ICD-10-CM M54.3, M54.4) due to lumbar disc herniation lasting more than 12 weeks, were included. Patients with disc herniation larger than size-2 or size-3 according to the MSU Classification were eligible for participation. Intervention was divided in two groups. In Group 1, patients underwent microdiscectomy and Interspinous Dynamic Stabilization System (IDSS). Meanwhile, in Group 2, patients received discectomy and posterior lumbar interbody fusion (PLIF). The primary outcome measure was the length of stay and costs during hospital admission. We also evaluated several other outcome parameters, including 90- day readmission rate, 90-day complication rate, and re-operations rate. The study was an observational prospective cohort study carried out from January 2015 to August 2016 in which two surgical techniques were compared. Our hypothesis was that a less aggressive procedure, such as discectomy and DSS, will decrease the length of stay and costs, and that it will also reduce the rate of complications with respect to PLIF.

**Results:**

A total of 67 patients (mean age 39.8 ± 8.4 years) were included. Patients in the PLIF group had a length of stay increase of 109% (4.52 ± 1.76 days vs 2.16 ± 1.18 days *p* < 0.001) and an in-hospital cost increase of 71% (1821.97 ± 460.41€ vs. 1066.20 ± 284.34€ p < 0.001). The reduction of one day of stay is equivalent to a reduction of total in-hospital costs of 12.5%. Patients in the IDSS cohort had no significant differences regarding PLIF cohort in the 90-day readmission rate (12.9% vs 11.1% € *p* > 0.999, respectively), 90-day re-operation rate (12.9% vs 11.1% € p > 0.999) and 90-day complication rates (35.5% vs 52.8% € *p* > 0.156). Dural tear and urinary tract infection rates were higher in the PLIF cohort (13.9% vs 3.2%. *p* = 0.205 and 11.1% vs 0% *p* = 0.118, respectively). Implant related complications were the most frequent in both IDSS and PLIF groups (32.3% vs 38.9% *p* = 0.572).

**Conclusions:**

Patients who underwent IDSS had a significant decrease of the length of stay and costs in relation to PLIF group. No significant differences were found in 90-day readmission and reintervention rates for both groups. Although differences were not significant, dural tear and urinary tract infection rates were lower in the interspinous group. IDSS or PLIF after discectomy, did not protect against subsequent 90-day re-operation or readmission compared to discectomy alone.

## Background

Low back pain and sciatica due to lumbar disc herniation has become a major public health problem [1]. The prevalence of symptomatic herniated lumbar disc is about 1–3%, with the highest prevalence among people aged 30–50 years. [2] Annually, it is estimated that 2.75 out of 1000 people with episodes of low back pain will suffer an episode of hospitalization [3]. Along with this, the number of lumbar spine surgeries has been increasing during the last 20 years, which also leads to an increase in hospital costs and complications related to surgery [4, 5]. Traditionally, the surgical treatment of the lumbar disc herniation has been discectomy [6]. However,although relief of sciatica, postoperative low back pain persist in some cases and iatrogenic instability following lumbar discectomy can lead to a reoperation. Reoperation rates of 12–14%% have been reported [7, 8]. To prevent this complication, discectomy associated with posterior lumbar interbody fusion (PLIF) has been used [9–11] Some authors report better results with fusion than with discectomy alone [12], while others state that fusion is rarely indicated because satisfactory results can be obtained by disc excision alone [13, 14]. A less aggressive technique between discectomy and fusion may be the discectomy associated with an interspinous dynamic stabilization system (IDSS) [15–17].The evidence whether to perform a PLIF or an IDSS in association with disc excision remains controversial and inconsistent with low number of cases. Therefore, no definitive conclusions could be made. Safe surgery, according to recent surgical safety strategies, is related to the occurrence of adverse events after the surgery and the rate of reoperation (related to initial procedure) in the first 90 days [18, 19]. IDSS are characterized by less invasiveness compared to PLIF, thus, it seems to offer higher surgical safety in terms of early postoperative ambulation decreasing medical complications rates and surgical wound infection rate [20]. Additional advantages of a less invasive procedure could be the reduction of hospital stay and consequently the reduction of hospital cost [21].

The present study was undertaken to further clarify whether the intervention (IDSS vs. PLIF) associated with discectomy modifies the length of stay (LOS) and in-hospital costs, in patients with lumbar disc herniation,. A secondary goal was to determine how these surgical procedures modify 90-day readmission, reintervention and complication rates.

## Methods

### Study design

This is an observational prospective cohort study including patients with lumbago / sciatica (ICD-10-CM M54.3, M54.4 / ICD-9-CM 724.3) due to lumbar disc herniation (Intervertebral disc disorder with myelopathy, lumbar region (ICD-10-CM M51.06 / ICD-9-CM 722.73) [22, 23] All patients were operated in a single institution from January 2015 to August 2016.

Our hypothesis is that the patients undergoing discectomy plus IDSS will have a decrease in the mean LOS and costs compared to that obtained with discectomy plus PLIF. A less aggressive procedure such as discectomy plus IDSS will also decrease the rate of surgical complications with respect to discectomy plus PLIF.

### Inclusion criteria

**Fig. 1:**
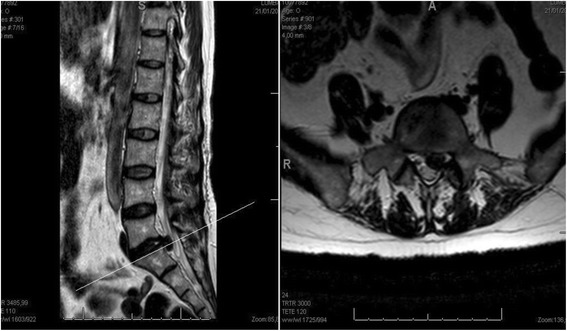
Rm T2. Sagital and Axial. Patient with 1 level lumbar disc herniation. Included either for group 1(discectomy and interspinous) or group 2 (discectomy and PLIF)

Voluminous 1–2 level lumbar disc herniation, (size-2 or size-3 herniations according to the MSU Classification) [24]. (Fig. 1) Age 18–50 years, body mass index (BMI) 18.5–35.0 kg/cm2, failure of nonoperative management for 12 weeks. Exclusion criteria included presence of previous lumbar spine surgery, spondylolisthesis, scoliosis greater than 10 degrees and degenerative disc changes Pfirman grade 4 or 5 (collapse of the disc space). [25, 26].

### Operative techniques

The first cohort included all patients who underwent open discectomy with limited disc excision using microscope assisted surgery (ICD-10-PCS 0SB20ZZ / ICD-9-CM 80.50, 80.51) [22, 23]. After disc excision, the In-Space Synthes® implant was mounted underneath the supraspinous ligament. The second cohort consisted of patients who underwent open wide discectomy (ICD-10-PCS 0SB20ZZ / ICD-9-CM 80.50, 80.51) followed by posterior lumbar interbody fusion (ICD-10-PCS 0SG3071, ICD-10-PCS 0SG30Z1, ICD-10-PCS 0SG0071 / ICD-9-CM 81.07) with autologous tissue substitute [22, 23].

All individual participants gave their informed consent to be included in the study and the trial was approved by the Hospital Ethics Committee.

### Demographic evaluation

In both groups, demographics (sex, age), BMI, tobacco consumption, and Charlson comorbidity index (CCI) were recorded. [27]

### Surgical safety

The 90-day complication rates were assessed with the following data:

*Major medical complications:* such as mortality, respiratory failure (pneumonia or unplanned reintubation), pulmonary embolism, acute renal failure, myocardial infarction, and cerebrovascular accident.

*Minor medical complications:* including deep venous thrombosis, urinary tract infection, peripheral nerve injury, and ileus.

*Surgical complications*: including cerebrospinal fluid leakage, seroma, wound infection and dehiscence rates and post operative anemia <8 g/dl were also assessed.

### Implant related complications

**Fig. 2:**
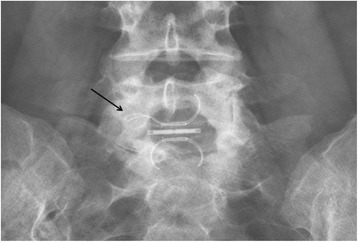
Lumbar spine x ray ap view. L5-S1 Interspinous complication, open anchorage wing. (black arrow)

**Fig. 3:**
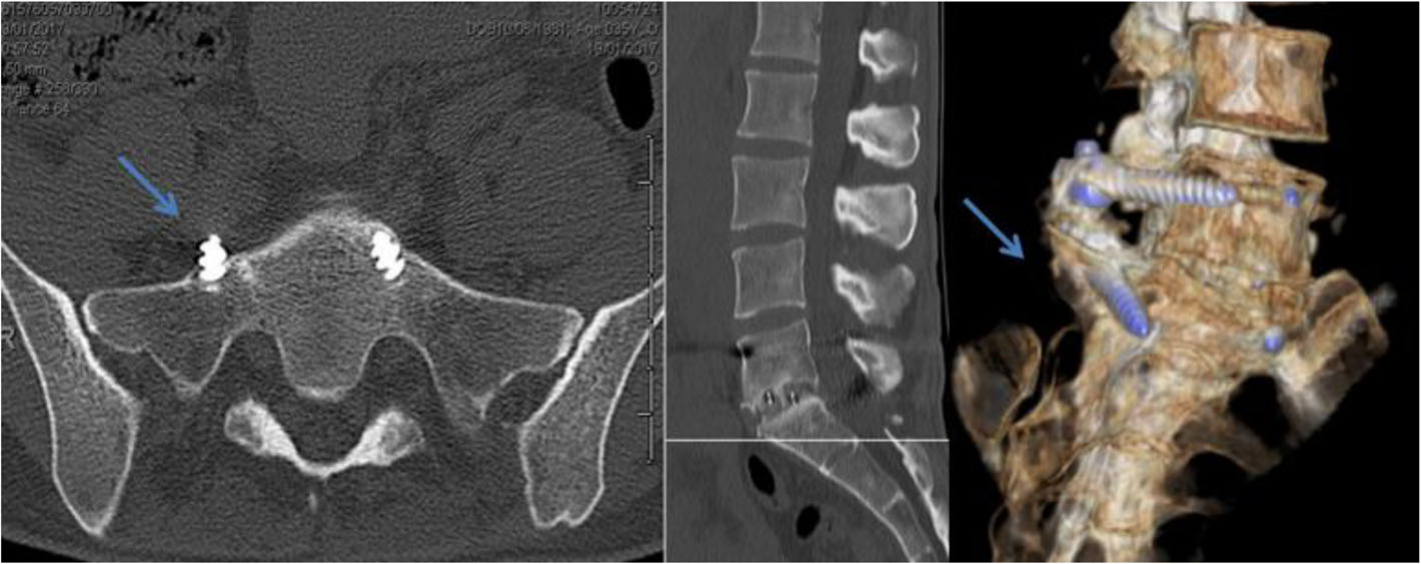
Lumbar CT scan. L5-S1 Fusion complication, anterior cortical perforation of vertebral body. S1 right side (blue arrows)

In the interspinous group, the implant was evaluated in anterior and lateral radiographs. Anchorage wings complications, rupture and dislocation were recorded (Fig. 2). In the PLIF cohort, pedicle screw misplacement was assessed. Five types of misplacement were recorded, namely medial cortical perforation (MCP), lateral cortical perforation (LCP), anterior cortical perforation of vertebral body (ACP), endplate perforation (EPP), and perforation of neural foramen (FP). Screw misplacement was considered positive, if screw violation was greater than 2 mm misplacement, and negative if screws were fully contained into the pedicle. [28] Computed tomography (CT) and radiographs were performed.(Fig. 3). The presence of a radiolucent rim of 1 mm or more surrounding the screw which is framed by radio-optic dense bone “double halo signal “in both radiographs projections, was considered as screw loosening. [29]

### Incidence of 90 day re-operation

Re-operation is considered as a bad outcome and therefore used as an outcome measure. The incidence of spinal re-operation in both groups was measured. In addition, 90 -day readmission rates due to any reason were calculated.

### Length of stay (L.O.S) and in-hospital costs

Data were obtained from digital history. Costs during hospital admission were calculated by an electronic hospital billing system. In order to asses only the cost during hospital admission, the price of the interspinous device in group 1(1500€) or the price of the pedicle screws, cage and rods in group 2 (3000€) were excluded. The following items per patient were added: 1) Human resources spent on surgery (Surgeon, anesthesiologist, nurses). 2) Expenses in surgical or medical material (serums, gauze, sutures, drainages, medication) 3) The cost spent on the stay (laundry, catering) 4) Complementary tests performed during the stay(for example, blood tests, x rays, etc). Knowing the value of these items, the individual costs of all operated patients in both groups were calculated.

### Statistics

Student’s t tests were used to compare L.O.S and in-hospital costs between groups, whereas the chi-square and exact Fisher test were used to compare complications and readmission rates, and also, to compare proportions of patients in each cohort with given demographics or comorbid conditions. To estimate the association between categorical variables and the intervention groups the odds ratio (OR) and its 95% confidence interval (CI) was calculated. Through multivariate analysis (Binary Logistic regression and Multiple Linear regression) confounding factors were controlled. For all analyses significance was set at *p* < 0.05.

Sample size: Based on our primary outcome, to detect a mean difference of 1 day in the mean LOS with a statistical power of 0.80, and a two-sided alpha of 0.05 a sample size of 28 patients per group was calculated. Based on previous studies a standard deviation of 0.5 days for IDSS group and 1.8 days for PLIF group was assumed for sample size calculation [30, 31].

## Results

### Patient demographics and Comorbidities

The present study included 67 patients with lumbar disc herniation. No patient was lost during follow-up. In Group 1, 31 patients underwent lumbar discectomy plus IDSS, meanwhile in Group 2, 36 patients received lumbar discectomy plus PLIF. All assessed demographics and comorbidities are summarized in Table 1. There were no significant differences between groups in terms of mean age and mean BMI. Proportions of females, smokers, and comorbidities were similar in both groups. In group 1 a higher proportion of single- instrumented level was found. A more detailed profile of CCI by groups is described in Table 2.

**Table 1:** Demographic and Co-Morbidity profile

	Group 1 (Interspinous)	Group 2 (Fusion)	*p*-value
Total Number (*n* = 67)	31	36	
Demographics			
Age^a^	38.2 (±9.3)	41.2 (±7.4)	0.144
Female^b^	13 (41.9%)	19 (52.8%)	0.376
BMI^a^	26.1 (±3.6)	26.5 (±3.8)	0.566
Smoking^b^	13 (41.9%)	13 (36.1%)	0.625
Charlson comorbidity index scoring^b^			
CCI = 0	25 (80.6%)	30 (83.3%)	0.775
CCI ≥ 1	6 (19.4%)	6 (16.7%)	
Instrumented levels^b^			
1 level	25 (80.6%)	18 (50%)	0.009
2 levels	6 (19.4%)	18 (50%)	

^a^Mean and standard deviation

^b^Number of patients and percentage by groups

**Table 2:** Charlson comorbidity index- clinical conditions by groups^a,b^

Comorbidity clinical conditions	Group 1 (Interspinous)	Group 2(Fusion)
Total Number (*n* = 67)	31	36
Peripheral vascular disease	1 (3.2%)	0
Chronic pulmonary disease	1 (3.2%)	1 (2.8%)
Mild liver disease	1 (3.2%)	0
Diabetes without complication	2 (6.5%)	2 (5.6%)
Tumors	1 (3.2%)	3 (8.3%)

^a^The following clinical conditions were not present: Myocardial infarct, Congestive heart failure, Cerebrovascular disease, Dementia, Connective tissue disease, Ulcers, Diabetes with complications, Leukemia, Paraplegia or Hemiplegia, Moderate or severe Renal disease, Lymphoma, Moderate or severe liver disease, Malignant Tumor. Metastasis. Acquired immune deficiency syndrome (AIDS)

Number of patients and percentage by groups

^b^Number of patients and percentage by groups

### Length of stay, 90-day re-admission rates, 90-day re-operations and in- hospital costs

Length of stay was significantly lower in the interspinous group when compared to fusion group (2,16 ± 1.18 days vs. 4.52 ± 1.76 *p* < 0.001). Costs were significantly lower in interspinous group as compared to fusion patients (1066.20 ± 284.34 € vs. 1821,97 ± 460.41 € *p* < 0.001 respectively.). Interspinous cohort had no significant less 90-day re-admission and 90-days re-operation rates compared to the fusion cohort (OR 1.18 95% CI: 0.27–5.19 *p* > 0.999 and OR 1.16 95% CI: 0.07–19.46 p > 0.999 respectively). LOS represented 27% (282.62 € / 1066 €) of the total in-hospital cost in the IDSS group and 31% (575.07 € / 1821.97 €) in the PLIF group. In-Hospital data concerning length of stay, in-hospital costs, re-admission and re-operations rates, are summarized in Table 3.

**Table 3:** Length of stay, In- hospital costs, 90 day re-admission and re-operations rates and Interspinous vs fusion group

	Group 1(Interspinous)	Group 2 (fusion)	*p*-value
Total Number (*n* = 67)	31	36	
Length of Stay^a^	2.16 (±1.18) days	4.52 (±1.76)days	< 0.001
Total In-Hospital Costs^a,b^	1066.20 (±284.34) €	1821.97 (±460.41) €	< 0.001
The stay cost (laundry, catering).	288.62 (±154.25) €	575.07 (± 227.09) €	< 0.001
Complementary tests cost	4.92 (±7.21) €	19.08 (±49.32) €	0.121
Human resources cost	596.67 (±158.56) €	937.29 (±190.32) €	< 0.001
Surgical or medical material cost^b^	175.98(±109.30)€	290.52 (±201.10) €	0.006
90 Days Readmission^c^	4 (12.9%)	4 (11.1%)	>0.999
90 Days Re-operation^c^	1 (3.2%)	1 (2.8%)	>0.999

^a^Mean and standard deviation

^b^Implant price, interspinous device (1500€) or PLIF pedicle screws and cage (3000€)not included

^c^Number of patients and percentage by groups

### 90-day medical complications, surgical complications and implant related complications

Interspinous group patients had no significant differences in the rate of total complications in regard to fusion group patients (OR 0.67 CI: 0.38–1.18 *p* = 0.156) Table 4. In the fusion group a non significant higher rate of urinary tract infection and dural tear was observed (OR 1.96 CI: 1.54–2.51 *p* = 0.11and OR 1.64 CI: 1.06–2.53 *p* = 0.2 respectively). Regarding to the implant related complications, the interspinous group had no significant lower rate respect to the fusion group. (OR 0.85 CI: 0.48–1.49 *p* = 0.57). In group 1 the most frequent implant related complication was anchorage wings complications (22.6%), followed by implant rupture (6.5%). In group 2, was the anterior cortical perforation of vertebral body (27.7%) (Figs. 2 and 3). No major medical complications were found in any of the 2 groups.

**Table 4:** 90-days medical complications, surgical complications and implant related complications. Interspinous vs fusion group

		Group 1(Interspinous)		Group 2 (Fusion)		*p*-value
Patients (*n* = 67)^a^		31		36		
Any complication		11(35.5%)		19 (52.8%)		0.156
Surgical Complications^b^		1(3.2%)		6(16.7%)		0.113
Dural tear		1 (3.2%)		5 (13.9%)		0.205
Seroma		0		1(1.5%)		>0.999
Implant Related Complications		10(32.3%)		14 (38.9%)		0.572
		Anchorage wings complications	7(22.6%)	Anterior screw	10(27.7%)	
		Broken implant	2 (6.5%)	Lateral screw	3 (8.3%)	
		Dislocated implant	1 (3.2%)	Broken screw	1(2.8%)	
				Screw loosening	0 (0.0%)	
Medical complications^c^						
Minor						
	Urinary tract infection	0		4 (11.1%)		0.118

^a^Number of patients and percentage by groups

^b^There was no case of Fistula, Wound infection, Wound dehisence, Nerve injury, Blood transfusion

^c^There was no case of Myocardaial Infarction, Pulmonary embolism, Cerebrovascular accident, Acute Renal Failure, Mortality, Deep venous thrombosis, Ileus

### Multivariate analysis

A more thorough assessment of the association between intervention (interspinous vs. fusion) and complications, LOS, and costs, was performed via multivariate analysis. It included other variables such as: Instrumented levels, age, sex and CCI. These analysis showed that the interspinous group was independently associated with lower length of stay −2.36 days (95% CI: -3.11-1.62 *p* < 0.001), lower costs −755.77 € (95% CI: -983.12 -565.59 p < 0.001) and had no significant differences in the rate of total complications in regard to fusion group OR 0.51 (95% CI: 0.16–1.6 *p* = 0.25).

## Discussion

Due to the increase in surgical procedures performed in patients with degenerative disc disease, there is a greater concern about the safety of the techniques and implants used [4]. Our study found no major complications, including death, in either group. In this sense, the reviewed literature reports more medical complications in fusion techniques than in interspinous surgical procedures. Puvanesarajah et al. reported up to 11.2% of major medical complications, 10% of urinary tract infection and 1.4% of deep venous thrombosis, after 1–2 lumbar spinal fusion surgery [18]. Whereas in a recent interspinous dynamic stabilization systematic review Leet al, reported no complications such as myocardaial infarction, pulmonary embolism, cerebrovascular accident, acute renal failure, mortality, deep venous thrombosis, ileus or urinary tract infection in patients who underwent interspinous dynamic stabilization surgery ^.32^ Regarding major or minor medical complications in our study, both techniques proved to be safe.

The most frequent complications were implant related complications in both groups. On the one hand, studies on IDSS,such as Diam, Wallis, and Coflex; reported an implant related complication rate up to 32.3% [32]. On the other hand, Gelais et al. in a systematic review, reported that rates of screw malposition in fusion surgery, vary considerably, they found the percentage of screws fully contained in the pedicle ranged from 28 to 85% [28]. In our study, we must highlight the rupture of an anchoring wing of the In-Space Synthes® device This complication has to be monitored, since if the 4 anchorage wings were ruptured, it could cause intracanal migration of the implant through the laminotomy site. In the fusion group, the most frequent alteration was the slight anterior protusion of the screw at S1vertebral body. We must take into account that anterior cortical penetration during sacral screw insertion carries a risk of neurovascular injury [33, 34]. Foxx et al., found 33 screws in contact with a major vessel in 182 patients and none of them suffered symptoms or sequelae [35]. In our patients, the majority of the implant related complications did not require clinical treatment or significantly affected treatment outcomes. The dural tear rate was lower in IDSS cohort than fusion cohort (3.2% and 13.9% *p* = 0.2 respectively). This agrees with what has been published in other studies, where the incidence in lumbar decompressive surgery varies widely (1–17%) and in general increases with the complexity of the spinal procedures performed [36, 37].

One of the main concerns after a discectomy is the need for reintervention. In this sense with the addition of IDSS or fusion after discectomy our reintervention rates (3.2% in IDSS and 2.8% in fusion group) are similar to those published with discectomy alone. Heinddel et al. revealed a rate of additional lumbar surgeries following single-level discectomy of 3.9% within 3 months, and 12.2% within 4 years [7]. Our short-term results revealed that the addition of IDSS or fusion does not protect against reintervention regarding simple discectomy. There are studies that reported even a higher percentage of reintervention in patients with lumbar spinal stenosis operated with decompression plus IDSS compared to traditional decompressive surgery [38–41] In addition, to know if adding fusion reduced the reintervention rate in relation to the decompression alone a previous randomized controlled trial had been performed dividing patients into 3 groups: decompression surgery, decompression plus posterolateral fusion, and decompression plus transforaminal interbody fusion. Revision surgery was performed in 3 patients, one in each group [42]. In this sense, one of the limitations of our study is not to have a control group with discectomy alone, to analyze if the reintervention rate varies with the addition of IDSS or fusion.

From a socioeconomic perspective, possibly the most significant finding of this study involved comparison of length of stay and in-hospital costs between the 2 groups. Patients in the fusion group had a length of stay increase of 109% compared to the interspinous group and an in-hospital costs increase of 71%. This result in which fusion increases length of stay and costs has been observed in previous investigations [42]. Hallett et al. found that posterolateral fusion increased 100% of the average stay (2 to 4 days) and 43% of costs (6617 to 9490 pounds) compared to decompression. A more detailed analysis of the costs showed that the catering and laundry expenses accounted for 27% of the total in-hospital cost in the IDSS group and 31% in the Fusion group.

The length of stay within our two study groups was similar to that published in other studies where the LOS ranged from 1.08 to 5.92 days [30].. The shortening of one day of stay resulted in a 12.5%.reduction of total hospital costs, these outcomes varied widely from those of Taheri et al., where it is reflected that the shortening of one day of stay had only a 3.4% effect on total costs [43].

IDSS cohort and PLIF cohort had similar 90 day readmission rate (12.9% and 11.1% respectively). This is a readmission rate higher than those of Modhia et al., who reported 9.7% and 7.2% readmission rate, and Bernatz JT et al., who reported a 30-day readmission rate between 4.2% and 7.4% [44, 45]. In this sense, our study reflected that IDSS or fusion did not protect against subsequent 90 day readmission rate compared to decompression alone.

### Limitations

The 90 days follow-up is sufficient to calculate variables such as, the stay, the cost, and the perioperative complications that happen within the first 90 days after surgery. Although, some long term complications related to the implant could be underestimated.

Another limitation of our study is the absence of a third control group with microdiscectomy alone. Therefore, we recommend studies that compare the 3 surgical techniques to verify if the instrumentation with interspinous device or fusion brings some benefit with respect to microdiscectomy alone. Due to ethical considerations allocation to intervention groups was not randomized. However, the resulting groups were similar in almost all the main characteristics which minimized possible biases. In addition, the use of multivariate analysis allowed us to control any confounding factors that could modify the association between intervention (interspinous or fusion) and complications, LOS, and costs.

## Conclusion

Patients who underwent microdiscectomy and interspinous dynamic stabilization system reduced their hospital stay and the in hospital costs, and there were no significant differences in the rate of total complications in comparison to PLIF group. These results support that the Fusion does not give benefits regarding microdiscectomy-IDSS in terms of surgical safety, complications, costs and readmissions in the 90 days after surgery. Interspinous dynamic stabilization or fusion after discectomy did not protect against subsequent 90-day readmission or reintervention compared to discectomy alone.

## Abbreviations

ACP: Anterior cortical perforation of vertebral body
BMI: Body mass index
CCI: Charlson comorbidity index
CI: Confidence interval
CT: Computed tomography
EPP: Endplate perforation
FP: Perforation of neural foramen
ICD-10-CM: International classification of diseases, tenth revision, clinical modification
ICD-9-CM: International classification of diseases, ninth revision, clinical modification
IDSS: Interspinous dynamic stabilization system
LCP: Lateral cortical perforation
LOS: Length of stay
MCP: Medial cortical perforation
OR: Dds ratio
PLIF: Posterior lumbar interbody fusion
